# Association of *Bacillus subtilis* and *Bacillus amyloliquefaciens*: minimizes the adverse effects of necrotic enteritis in the gastrointestinal tract and improves zootechnical performance in broiler chickens

**DOI:** 10.1016/j.psj.2023.103394

**Published:** 2023-12-19

**Authors:** Romário A. Rodrigues, Leandro A.M. Silva, Heloisa C. Brugnera, Natália Pereira, Mariana F. Casagrande, Lilian C. Makino, Caio R.S. Bragança, Rubén Pablo Schocken-Iturrino, Marita V. Cardozo

**Affiliations:** ⁎ Department of Reproduction Pathology and One Health, School of Agricultural and Veterinary Sciences, São Paulo State University (UNESP), Jaboticabal, São Paulo, Brazil; † Department of Fisheries and Aquaculture Resources, School of Agricultural Sciences of Vale do Ribeira, São Paulo State University (UNESP), Registro, São Paulo, Brazil; ‡ Laboratory of Microorganism Physiology, Department of Biomedical Sciences and Health, Minas Gerais State University, Passos, Minas Gerais, Brazil

**Keywords:** *Bacillus* spp., coccidiosis, growth performance, gut function, one health

## Abstract

This study aimed to evaluate the efficiency and capacity of the probiotic composed of *Bacillus subtilis* and *Bacillus amyloliquefaciens*, in improving the zootechnical performance of broiler chickens challenged with *Eimeria* spp. and *Clostridium perfringens*. The broilers were distributed in a completely randomized design in poultry isolators (12 birds each), resulting in 3 treatments: T1 (control, no challenge and no *Bacillus* in diet), T2 (challenged with *Eimeria* spp., followed by *Clostridium perfringens* infection and no *Bacillus* in the diet), and T3 (challenged with *Eimeria* spp., *Clostridium perfringens* and treated with *Bacillus subtilis* and *Bacillus amyloliquefaciens*). They were evaluated for a period of 29 d, divided into preinitial (1–7 d of age), initial (8–21 d), and growth (22–29 d) phases. Assessments of body weight, weight gain, feed consumption, and feed conversion were conducted, along with the classification of the scores and optical microscopy of the tract gastrointestinal. The animals challenged and treated with the probiotic containing *Bacillus* spp. showed improved indicators of zootechnical performance. Additionally, the animals challenged and treated (T3) had a better score for intestinal lesions compared to the other treatment groups. Therefore, the probiotic consisting of *Bacillus subtilis* and *Bacillus amyloliquefaciens* could be considered an effective option for disease prevention and improving the zootechnical performance of broiler chickens

## INTRODUCTION

Probiotics are defined as live microorganisms that, when consumed in the correct quantity, act beneficially in the host intestine, improving the balance of the intestinal microbiota (Fuller, 1989). Mostly, they are bacteria belonging to the *Lactobacillus* genus, capable of colonizing different parts of the intestine, ensuring benefits such as immune response modulation and enhanced nutrient absorption (Khan et al., 2020; Hashemi et al., 2023). The main mechanism of action of probiotics is competitive exclusion—when bacteria compete for adhesion sites in the intestinal epithelium, through the production of metabolites such as short-chain organics, bacteriocins and hydrogen peroxide, controlling the growth and the competitiveness of pathogens (Khalid et al., 2022), but also, there is an increase in their adhesion to the host mucosa, promoting better nutrient absorption and stimulation of the immune system (Yousefi et al., 2018; Ningsih et al., 2023).

In addition to the species belonging to the genus *Lactobacillus*, various microorganisms can function as probiotics, such as strains of the genus *Bacillus, Enterococcus, Bifidobacterium*, and even some yeasts and molds, like *Saccharomyces* (Tang and Lu, 2019; Khan et al., 2020). These microorganisms can provide benefits to hosts, as they are tolerant to the physical and chemical conditions of the gastrointestinal tract, such as pH and enzymatic activities, further producing growth-controlling metabolites of pathogens (Yousefi et al., 2018). The challenges lie in the association of different strains in the production of probiotics that have the ability to enhance production, improve animal health, and minimize risks to public health (Abd El-Hack et al., 2020).

In animal production, the use of antimicrobials as growth promoters, in addition to prophylaxis, accounts for approximately 73% of the total global consumption of these drugs (Van Boeckel et al., 2019). This situation is a matter of concern, as these subtherapeutic doses and antimicrobial residues in food may contribute to selective pressure on bacteria resulting from environmental contamination and food consumption, thus being a serious public health issue, driven by the selection of etiological agents resistant to drugs used in both human and animal use (Innes et al., 2020; Dréano et al., 2022; Talat et al., 2023). As a result, some countries in the European Union have initiated the prohibition of these compounds in livestock farming, requiring the adoption of viable and natural alternatives for replacement, without causing economic and social losses (Abd El-Hack et al., 2022).

In view of this, probiotics have been emerging as an option in the production of livestock animals aimed at reducing public health issues, while also contributing to antimicrobial-free practices (Ritzi et al., 2016; Lu et al., 2020). The use of probiotics in poultry production contributes to the establishment of a healthy intestinal environment, resulting in increased length of intestinal villi, allowing greater nutrient absorption and, consequently, improving the zootechnical performance. In the literature, numerous studies focus on microorganisms, evaluated individually or in association with other species, that possess ideal characteristics as probiotics. *Bacillus* spp. stand out among them due to their ability to form spores and resist various temperature and pH conditions, enabling their survival during feed purification and processing. Additionally, they play a significant role in the intestinal tract of birds (Jadhav et al., 2015; Ciurescu et al., 2020).

*Bacillus subtilis* is a facultative anaerobic bacterium, and in birds, it is capable of engaging in the production and secretion of various digestive enzymes and vitamins. This contributes to the digestion of nutrients and animal growth, while also playing a significant role in improving intestinal antioxidant levels (Bai et al., 2017). In the gastrointestinal tract, *B. subtilis* requires a substantial amount of free oxygen, which acts as a limiting factor for the colonization of other species, especially pathogenic ones. This condition favors the dissemination of anaerobic strains responsible for regulating the intestinal microbiota (Mohamed et al., 2022).

Similar to *B. subtilis, Bacillus amyloliquefaciens* promotes the secretion of digestive enzymes, enhancing the digestion and absorption of nutrients, contributing to animal performance and the suppression of *C. perfringens* (Tsukahara et al., 2018; Sun et al., 2022; Arshad Iqbal et al., 2023). Some authors suggest that this strain produces effective bacteriocins against pathogens and has the ability to reduce lipopolysaccharides (**LPS**). For these reasons, its use has been consistent in poultry feed processing (De Oliveira et al., 2019; Ahmat et al., 2021; Du et al., 2023). Both aforementioned *Bacillus* species have been employed in probiotic formulations, either alone or in combination with other strains with potential. Such formulations have demonstrated significant improvements in performance, immunity, reduction of lesions caused by necrotic enteritis, decreased mortality, bedding quality, among other benefits (Jadhav et al., 2015; Cirilo et al., 2023).

For these reasons, this study aimed to determine the efficiency and capability of the probiotic manufactured with *Bacillus subtilis* and *Bacillus amyloliquefaciens* in improving the zootechnical performance of broiler chickens challenged with the pathogens *Clostridium perfringens* and *Eimeria* spp. through zootechnical assessments and histopathological analysis of intestinal lesions.

## MATERIALS AND METHODS

### Experimental Design

A total of 36 male broiler chicks from the commercial Cobb 500 lineage, 1-day old, with an initial average weight of 40 g, were used in the study. The broilers were distributed in a completely randomized design in poultry isolators (12 birds each), divided into 3 treatments, with 12 animals per treatment, and each bird was considered an experimental unit (replication). The animals had access to water and feed “ad libitum” throughout the experimental period (1–29 d of age). The feed was formulated according to the nutritional requirements of each phase of bird rearing (prestarter: 1–7 d of age, starter: 8–21 d of age, and grower: 22–29 d of age) (Rostagno et al., 2017). The experimental protocol used in the current study was previously approved by the Ethics Committee of Animal Care and Use of the São Paulo State University “Julio de Mesquita Filho,” School of Agricultural and Veterinary Sciences, Jaboticabal, state São Paulo, Brazil.

The treatments were carried out in 3 isolators, each with a structure to prevent cross-contamination between the experiments, as follows: T1 (control: no challenge and no *Bacillus* in the diet), T2 (challenged: infection with *Eimeria* spp. on the 8th day of life, followed by *Clostridium perfringens* infection on the 14th day of life, and no *Bacillus* in the diet), and T3 (challenged and treated: infection with *Eimeria* spp. on the 8th day of life, followed by *Clostridium perfringens* infection on the 14th day of life, and with a probiotic containing *Bacillus subtilis* and *Bacillus amyloliquefaciens* in the diet).

### Challenge

The animals were challenged with *Clostridium perfringens* (ATCC 13124) and a suspension of *Eimeria* spp., which included: *E. acervulina, E. brunetti, E. maxima, E. necatrix, E. praecox, E. tenella*, and *E. mitis*. The inoculum of *C. perfringens* was seeded into modified brain heart infusion (**BHI**) broth with cysteine hydrochloride, previously sterilized, and incubated under anaerobic conditions for 48 h at 37°C. The inoculum concentration reached 10^8^ CFU/mL and was determined by spectrophotometry and then was stored at 4°C until the time of challenge. Each bird was orally inoculated with 0.5 mL of bacterial suspension, approximately 5 × 10^7^ CFU/mL. For *Eimeria* spp., an overdose of 0.2 mL per bird was administered orally using a coccidiosis vaccine.

### Probiotic

The probiotic is a product based on *Bacillus subtilis* and *Bacillus amyloliquefaciens*. The product was added to the feed at a proportion of 200 ppm of the probiotic in the diet for all rearing phases (prestarter, starter, and grower), resulting in a concentration of 1 × 10^7^ CFU/kg of feed for *B. subtilis* and 7.42 × 10^7^ CFU/kg of feed for *B. amyloliquefaciens*.

### Intestinal Lesions and Histopathological Analysis

Five birds of each treatment were slaughtered at 2 time points, 21 and 29 d of age. Subsequently, intestinal portions of the jejunum, ileum, and cecum were removed for visual inspection and assessment of the lesion scores, being classified as follows: absent (0), mild (1), moderate (2), and severe (3), based on the presence of inflammation, hemorrhage, and lymphoid reactivity in Peyer's patches, as represented in Table 1 (Johnson and Reid, 1970; Belote et al., 2019).

**Table 1:** Classification or scoring determination based on the lesion and the presence of specific factors in the intestinal mucosa and/or feces of birds from various treatments.

Value	Characteristics of the degree of lesion
0	Birds without macroscopic lesions
+1	Presence of small petechiae observed in the serosa of the middle intestine; there may be a small amount of orange mucus; no ballooning or thickening of the intestine
+2	Serosal surface painted with numerous petechiae; the intestine may be filled with orange mucus; slight ballooning and thickening of the intestine
+3	The intestinal wall is ballooned and thickened; rough mucosal surface; intestinal content with small clots
+4	Intestinal wall thickened and ballooned along almost its entire length; contains clots in the intestinal content
+5	Several authors consider a grade of +5 for birds that died as a result of the infection

Source: Johnson and Reid (1970).

After visual analysis, the intestinal samples were fixed in a 10% formalin solution buffered with disodium hydrogen phosphate (**Na_2_HPO_4_**) and sodium phosphate (**NaH_2_PO_4_**) (pH 7.2). After 24 h of fixation, the samples were sectioned and subsequently embedded in paraffin. For slide preparation, sections with a thickness of 5 µm were cut using a microtome (Leica Biosystems) and subsequently stained with hematoxylin and eosin. The slides were then evaluated using an optical microscope for analysis.

### Zootechnical Performance

To evaluate zootechnical performance, we employed an adaptation of the methodology described by Whelan et al. (2019). The following parameters were evaluated at the end of each phase (d 7, 21, and 29): feed intake per bird (g/bird), daily feed intake per bird (g/bird/d), weight gain per bird (g/bird), daily weight gain per bird (g/bird/d), and feed conversion ratio (g/g). For this purpose, the birds and the feed were weighed at the beginning and end of each phase, namely prestarter, starter, and grower phase. The mortality rate at each stage was recorded for the calculation of viability (%).

### Statistical Analysis

The data were analyzed using SAS software (SAS Institute Inc., Heidelberg, Germany). Initially, the presence of outlier data was assessed, and the normality of residuals was tested using the Shapiro-Wilk test and when this was not met, logarithmic transformation or analysis using the square root was applied as necessary. Subsequently, the Proc Mixed procedure was employed for mixed models.

Among the 15 different covariance structures tested, the one based on the lowest value of the corrected Akaike information criterion (**AICC**) was selected (Wang and Goonewardene, 2004). The model included fixed effects of treatment, which were separated using the Tukey test. A significance level of 5% was adopted for all the conducted tests.

## RESULTS

### Intestinal Lesions

The scores obtained throughout the 3 treatments are described in Table 2. As expected, in the gastrointestinal tract of animals in group T1 (control), there were no macroscopic lesions, and the animals remained healthy. In group T2 (challenged), the animals exhibited characteristic signs of infection, such as progression throughout the experiment, including apathy, reduced weight gain, and liquid feces. During necropsy, petechiae were observed in the external intestinal mucosa, extensive intestinal areas with orange coloring, a thicker serosal layer, distended intestinal loops due to gas, and yellowish foamy content in the intestinal lumen. In T3 (Challenged + Treated), the obtained score was mild on d 21 and moderate on d 29. Upon visual inspection, the bird appeared healthy, and there was a small amount of orange mucus in the intestinal lumen, without intestinal ballooning or thickening, and only a few petechiae. The intestinal mucosa showed a few areas of hemorrhage in the Peyer´s patches and small focal areas in the enteric mucosa, characteristic of necrosis.

**Table 2:** Intestinal lesion score of broiler chickens throughout the experiment.

Phase	Treatments		
	Control	Challenged	*Bacillus*
Prestarter	0	0	0
Starter	0	2	1
Grower	0	3	2

### Histopathological Analysis

**Figure 1:**
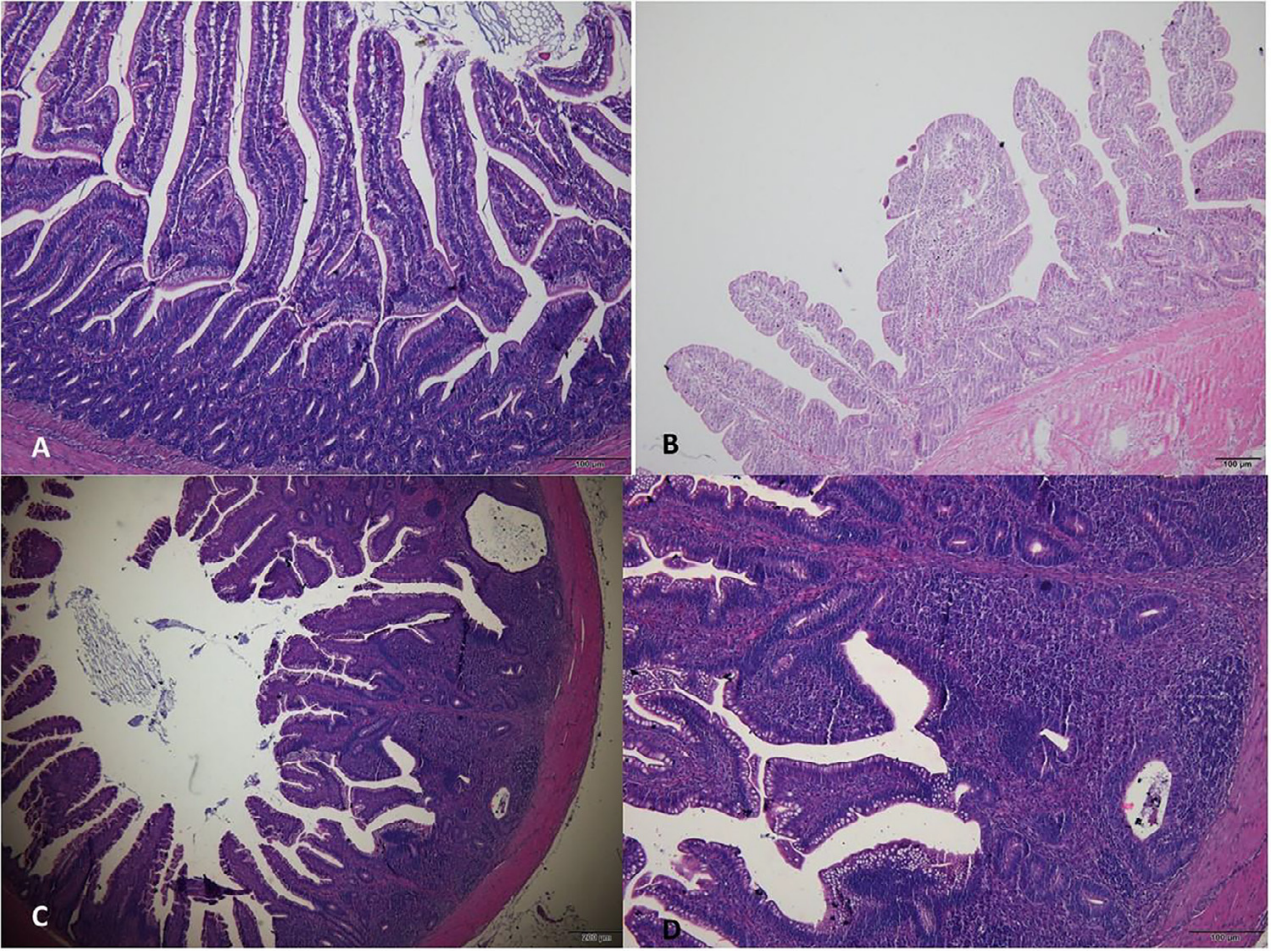
Photomicrographs of the intestine of birds in the control group (T1). (A) Jejunum (scale bar = 100 µm). (B) Ileum (scale bar = 100 µm). (C) Cecal tonsil (scale bar = 200 µm). (D) Magnified view of the cecal tonsil, showing details of lymphoid tissue (scale bar = 100 µm). Hematoxylin and eosin.

**Figure 2:**
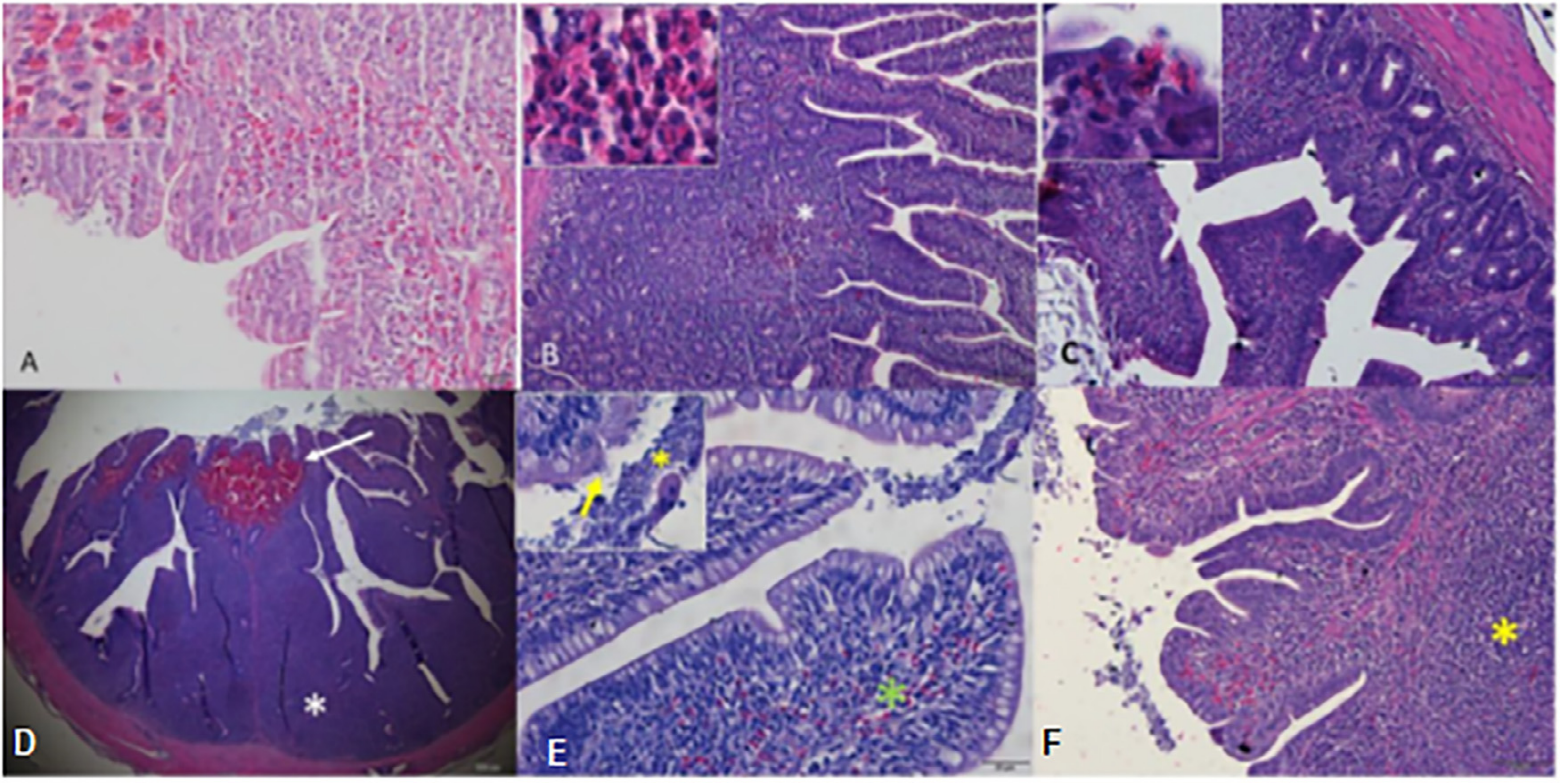
Photomicrographs of the intestine of birds in the group challenged with *Eimeria* spp. and *Clostridium perfringens* (T2). (A) Jejunum segment showing an area of villus hemorrhage (hemorrhage detail, scale bar = 20 µm). (B) Jejunum with an area of heterophilic infiltrate (*) in the intestinal mucosa (heterophils detail, scale bar = 100 µm). (C) Ileum with lymphocytic infiltrate in the mucosa associated with heterophils (heterophils detail, scale bar = 50 µm). (D) Cecal tonsil with an area of hemorrhage (arrow) at the apex of the villi. Notice the intense lymphoid reactivity (*, scale bar = 200 µm). (E) Apex of cecal tonsil villus with a focus of hemorrhage (*green) and presence of basophilic bacilli (bacteria) in the intestinal lumen (*yellow, detail) and adhered to the intestinal mucosa (arrow, detail, scale bar = 20 µm). (F) Cecal tonsil segment with pronounced inflammatory infiltrate in the mucosa (*scale bar = 50 µm). Hematoxylin and eosin.

In the histopathological analysis of group T1, no lesions were observed in the segments of the small intestine (jejunum and ileum) and large intestine (cecum), thus demonstrating intact intestinal mucosa (Figure 1). In group T2, however, it was observed the presence of a predominantly lymphocytic inflammatory infiltrate in all segments of the intestine, extending from the submucosa to the region beneath the epithelium lining the intestinal mucosa. This infiltrate was associated with the presence of heterophils and areas of hemorrhage at the apex of the villi and cecal tonsil, which were predominant during the period from 8 to 21 d. During the 22- to 29-day period, there was an intensification of inflammation in all intestinal segments, accompanied by the presence of basophilic bacilli indicating bacterial colonization adjacent to the luminal intestinal contents. Furthermore, bacterial clumps were observed adhering and causing microfissures in the lining epithelium of the intestinal mucosa (Figure 2), resembling the necrotic lesions caused by *Clostridium perfringens*, where the tissue appears hyperemic and with the presence of numerous inflammatory cells, typically heterophilic granulocytes (Benedet et al., 2021).

**Figure 3:**
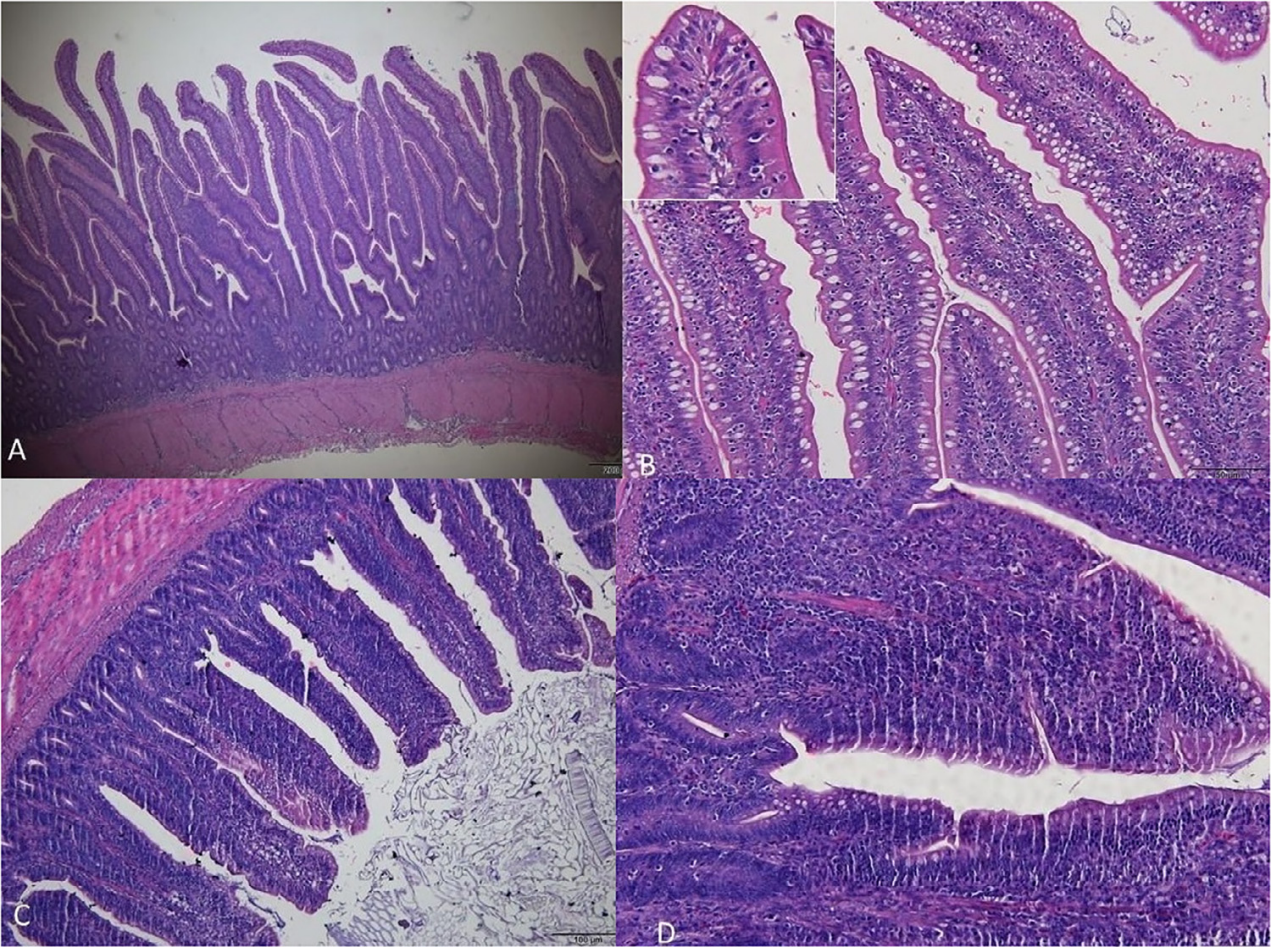
Photomicrographs of the intestine of birds challenged with *Eimeria* spp. and *Clostridium perfringens* and administered the probiotic (T3). (A) Jejunum (scale bar = 200 µm). (B) Jejunum with intact mucosa and presence of epithelial transmigration of leukocyte (detail, scale bar = 50 µm). (C) Ileum (scale bar = 100 µm). (D) Ileum with moderate lymphocytic inflammation in the intestinal mucosa (scale bar = 50 µm). Hematoxylin and eosin.

**Figure 4:**
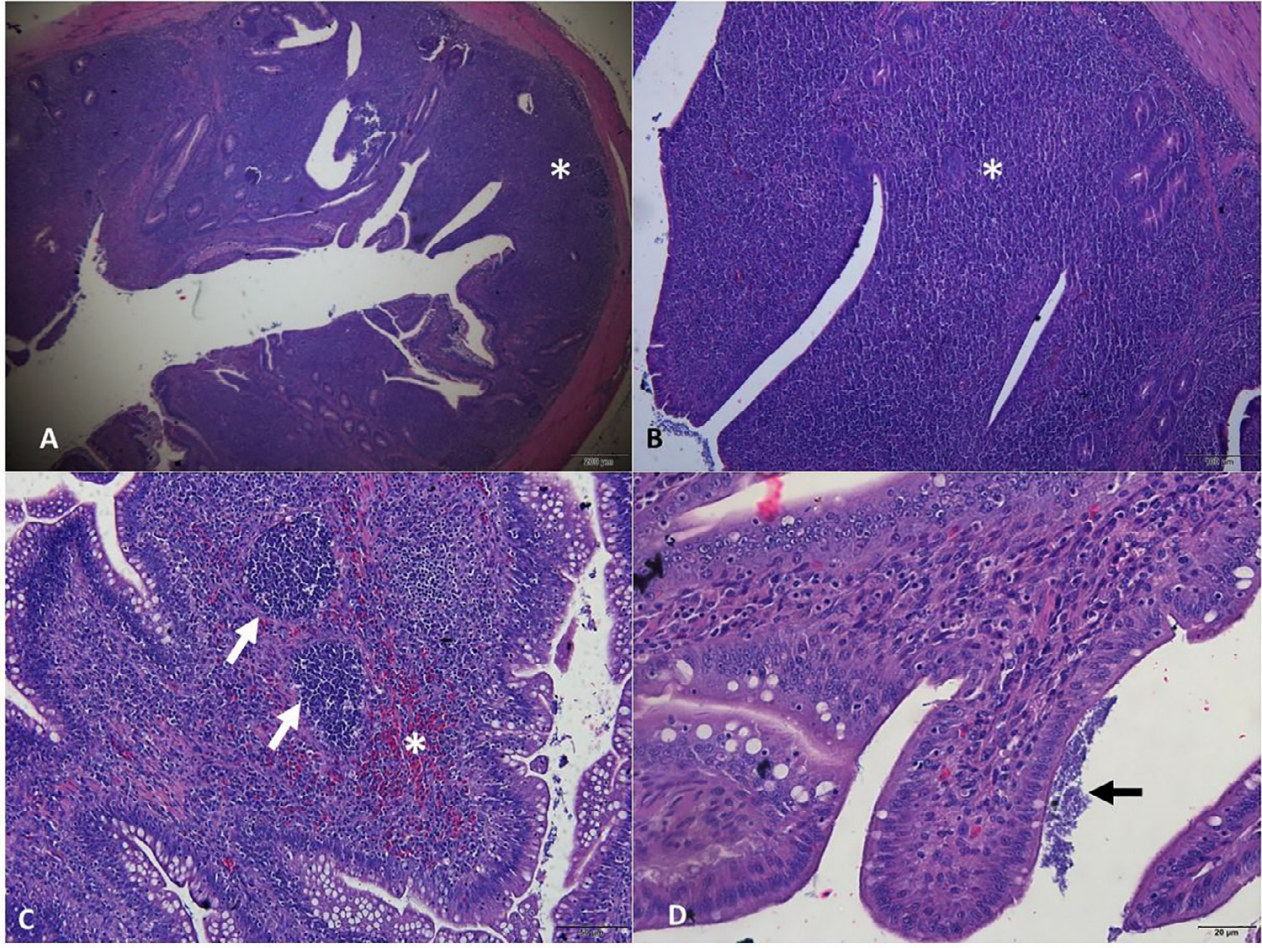
Photomicrographs of the intestine of birds challenged with *Eimeria* spp. and *Clostridium perfringens* and administered the probiotic (T3). (A) Cecal tonsil showing pronounced lymphoid reactivity (*; scale bar = 200 µm). (B) Detail of the reactivity shown in A (*; scale bar = 100 µm). (C) Lymphoid proliferation with follicular appearance in the cecal mucosa (arrows) and foci of heterophilic infiltrate (*; scale bar = 50 µm). (D) Bacterial clumps in the intestinal lumen (arrow), without causing damage to the epithelium lining the intestinal mucosa. Hematoxylin and eosin.

The histopathological analysis of group T3 revealed an intestinal inflammatory response to bacterial presence, albeit less pronounced than in group T2. In addition, no areas of hemorrhage caused by *C. perfringens* were observed (Figure 3). Adhesion of bacteria to the lining epithelium of the intestinal mucosa was discreet or absent, without the occurrence of microfissures. However, the lymphoid reactivity in the cecal tonsils was found to be more intense than in the T2 group. The frequent presence of trans-epithelial migration of leukocytes (i.e., the translocation of defense cells between epithelial layers) was observed, and the epithelium of the intestinal mucosa remained preserved in the birds (Figure 4).

### Zootechnical Performance

In the preinitial phase (1–7 d), no significant differences were observed in performance parameters—weight gain (**WG**), feed intake (**FI**), and feed conversion ratio (**FCR**)—among animals in different treatments (Table 3). In the initial phase (8–21 d), which included the challenge with *Eimeria* spp. and *C. perfringens*, there was a significant difference (*P* > 0.05) in WG between animals in T2 and T3, with the latter group showing the highest gain. FI and FCR were not affected by the treatments (*P* < 0.05) (Table 4). During the growth period (22–29 d), significant differences in animal weight (**AW**) were observed at the end of the phase when comparing groups T2 and T3 (Table 5). The analysis of animal performance throughout the experimental period can be seen in Table 6. It is noteworthy that the T1 group only showed statistical differences compared to the other groups (T2 and T3) in FCR, and the WG of animals fed the diet containing added *Bacillus subtilis* and *Bacillus amyloliquefaciens* was superior to the challenged animals.

**Table 3:** Broiler chicken performance during the preinitial phase.

Variables	Treatments		
	Control	Challenged	*Bacillus*
Initial weight (g)	40	40	40
Weight 7 d (g)	138	145	143
Weight gain (g)	97.5	105	103
Feed intake per bird (g)	83	97.5	92.5
Feed conversion (g/g)	0.85	0.93	0.9

**Table 4:** Performance of broiler chickens in the initial phase.

Variables	Treatments			
	Control	Challenged	*Bacillus*	*P* value
Weight 7 d (g)	138	145	143	-
Weight 21 d (g)	738.33^ab^	723.33^b^	785.00^a^	0.0413
Weight gain (g)	600.83^ab^	578.33^b^	642.00^a^	0.0394
Feed intake per bird (g)	1058.33	1066.67	1089.57	-
Feed conversion (g/g)	1.78	1.86	1.71	0.1378

**Table 5:** Broiler chicken performance during the growth phase.

Variables	Treatments			
	Control	Challenged	*Bacillus*	*P* value
Weight 21 d (g)	738.33^ab^	723.33^b^	785.00^a^	0.0413
Weight 29 d (g)	1445.71^ab^	1377.14^b^	1518.33^a^	0.066
Weight gain (g)	692.86	671.43	721.67	0.2099
Feed intake per bird (g)	1028.57	1300	1110	-
Feed conversion (g/g)	1.49^a^	1.94^b^	1.54^a^	<0.0001

**Table 6:** The performance of broiler chickens throughout the experiment.

Variables	Treatments			
	Control	Challenged	*Bacillus*	*P* value
Initial weight (g)	40	40	40	-
Weight 29 d (g)	1445.71^ab^	1377.14^b^	1518.33^a^	0.066
Weight gain (g)	1405.71^ab^	1337.14^b^	1478.33^a^	0.0066
Feed intake per bird (g)	2170.24	2464.17	2292.07	-
Feed conversion (g/g)	1.54^a^	1.84^b^	1.55^a^	0.0366

## DISCUSSION

In recent years, there has been an increase in the regulation of the use of growth-promoting antimicrobials in animal production. In the poultry scenario, enteritis and intestinal disorders have intensified with the removal of these drugs, and the adoption of alternatives that promote the intestinal health of birds is necessary to improve performance and immune response (Gadde et al., 2017). One major concern is necrotic enteritis (**NE**) due to its multifactorial nature and significant financial impact on production. This disorder is caused by *Clostridium perfringens*, which employs various virulence strategies, including metabolic enzymes and toxins that cause tissue degradation (Hernandez-Patlan et al., 2019; Daneshmand et al., 2022).

Therefore, probiotics have emerged as the predominant alternative to antimicrobials, thanks to their therapeutic action against pathogens and their role in enhancing both intestinal health and zootechnical performance in poultry (Park et al., 2020; Wang et al., 2021).

*Bacillus subtilis* exhibits the capacity to enhance body weight and feed efficiency. In animals afflicted with necrotic enteritis, this strain can lower mortality rates and decrease the intestinal lesion score, displaying effectiveness comparable to the use of antimicrobials (Ningsih et al., 2023). Furthermore, research involving this microorganism has showcased a substantial improvement in morphological aspects and an increase in the length of intestinal villi. This improvement coincides with a reduction in pathogenic bacteria in broilers. These observed effects highlight the strain's significance as a growth promoter, contributing to elevated immunoglobulin levels, improved intestinal health, and disease prevention in poultry (Jayaraman et al., 2013; Abd El-Ghany et al., 2022; Wang et al., 2023).

Another strain of significant importance is *B. amyloliquefaciens*, which displays a similar behavior in the intestinal microbiota by inhibiting the proliferation of *C. perfringens* through the disruption of *quorum sensing* mechanisms—a communication system among cells that induces the expression of genes involved in the function and metabolism of the etiological agent (Madigan et al., 2016; Whelan et al., 2019). Additionally, this strain has demonstrated a noteworthy capability to enhance nutrient absorption (Gharib-Naseri et al., 2021). However, there are reports suggesting that the established effects of this strain are substantial and do not show improvements in zootechnical performance and necrotic enteritis in broilers (Jerzsele et al., 2012; Geeraerts et al., 2016). Therefore, the present experiment aimed to evaluate the effect of the association between *Bacillus subtilis* and *Bacillus amyloliquefaciens* in the feed of broilers challenged with *Eimeria* spp. and *Clostridium perfringens.*

During the experiment, there was an expectation that the damages caused by the destruction of enterocyte cells by *Eimeria* spp., coupled with the toxin production of *C. perfringens*, which establishes itself in these necrotic regions, would worsen the performance of the birds in the initial phase. Given that *Eimeria* spp. infection induces changes in intestinal mucosa, making them susceptible to other pathogens (Abdisa et al., 2019). However, these differences were not statistically significant between the animals in groups T1 and T2.

The characteristics noted during the clinical and histological analysis of group T2 were in line with the profile of necrotic enteritis. In this scenario, broilers displayed lesions along the mucosa, mucus, and abnormal content in the intestine (De Oliveira et al., 2019) (Figures 1 and 2). Furthermore, there were changes in intestinal villi, exhibited altered and damaged characteristics, with reduced length and the presence of hemorrhages when compared to the control group (Jayaraman et al., 2013).

Upon evaluating group T3, the lesions found were less intense and of a lower degree when compared to the animals in group T2 (Figure 4). The response in the modulation of intestinal microbiota can be observed in the T3 group, due to the presence of leukocytes and lymphoid reactivity. Thus, it reinforces the effect of dietary supplementation with *B. subtilis* and *B. amyloliquefaciens,* as these are capable of assisting in the modulation of host's immune response, promoting an increase in antibody and the activity of defense cells in the intestinal mucosa. Therefore, the probiotic tested in the animal helped reduce pathogen infection and also preserved the integrity of the intestinal mucosa, reducing the degree of lesion scores.

Most studies showed positive effects of *B. amyloliquefaciens* on zootechnical performance, especially when it comes to variables like body weight, daily weight gain, average daily feed intake and feed conversion (Shini et al., 2020; Gharib-Naseri et al., 2021; Zhang et al., 2022). However, there are reports that these effects are substantial and do not show improvements in zootechnical performance and necrotic enteritis in broiler chickens (Jerzsele et al., 2012; Geeraerts et al., 2016). These results are in line with existing literature, as the positive effects of combining *Bacillus subtilis* and *Bacillus amyloliquefaciens* are better than when administered individually (Sandvang et al., 2021; Khalid et al., 2022; Zhang et al., 2022; Larsberg et al., 2023).

The main results were highlighted during the growth phase, which were consistent with the literature findings regarding broiler chickens challenged with *Eimeria* and *C. perfringens,* highlighting differences between the final weight of groups T2 and T3, the histopathological alterations, and the zootechnical performance (Tactacan et al., 2013). In this context, the present study highlights that when these strains are associated, they promote positive outcomes in terms of body weight, weight gain, and feed conversion, while also minimizing harmful effects on the intestinal mucosa (Angelakis and Raoult, 2010; Gao et al., 2017; Hernandez-Patlan et al., 2019).

Therefore, the probiotic composed of *Bacillus subtilis* and *Bacillus amyloliquefaciens* has proven to be an effective option for preventing diseases and improving the zootechnical performance of broilers.
